# Association of* lincRNA-p21* Haplotype with Coronary Artery Disease in a Chinese Han Population

**DOI:** 10.1155/2016/9109743

**Published:** 2016-06-02

**Authors:** Sai-sai Tang, Jie Cheng, Meng-yun Cai, Xi-li Yang, Xin-guang Liu, Bi-ying Zheng, Xing-dong Xiong

**Affiliations:** ^1^Institute of Aging Research, Guangdong Medical University, Dongguan 523808, China; ^2^Institute of Biochemistry & Molecular Biology, Guangdong Medical University, Zhanjiang 524023, China; ^3^Key Laboratory for Medical Molecular Diagnostics of Guangdong Province, Guangdong Medical University, Dongguan 523808, China; ^4^Department of Cardiovascular Disease, The First People's Hospital of Foshan, Foshan 528000, China; ^5^Institute of Laboratory Medicine, Guangdong Medical University, Dongguan 523808, China

## Abstract

lincRNA-p21 plays an important role in the pathogenesis and progression of coronary artery disease (CAD). To date, the biological significance of polymorphisms in* lincRNA-p21* on CAD risk remains unknown. Here we aimed to evaluate the influence of* lincRNA-p21* polymorphisms on individual susceptibility to CAD. Genotyping of four tagSNPs (rs9380586, rs4713998, rs6930083, and rs6931097) within* lincRNA-p21* gene was performed in 615 CAD and 655 controls. The haplotype analysis showed that the haplotype G-A-A-G (rs9380586-rs4713998-rs6930083-rs6931097) was statistically significantly associated with the reduced risk for CAD (OR = 0.78,* P* = 0.023). Stratified analysis revealed that G-A-A-G haplotype was at a significantly lower risk for myocardial infarction (MI) (OR = 0.68,* P* = 0.010). We also found that haplotype G-A-A-G had a more pronounced decreased risk for premature CAD or MI subjects (OR = 0.67,* P* = 0.017 for premature CAD, and OR = 0.65,* P* = 0.041 for premature MI, resp.). Our data provide the first evidence that the G-A-A-G haplotype of* lincRNA-p21* is associated with decreased risk of CAD and MI, particularly among premature CAD/MI in the Chinese Han population. Further studies with more subjects and in diverse ethnic populations are warranted to clarify the general validity of our findings.

## 1. Introduction

Coronary artery disease (CAD) is the world's leading cause of death [1]. Many risk factors have been demonstrated to conduce to the pathogenesis of CAD, including male gender, body fat, smoking, diabetes mellitus, hypertension, high serum cholesterol, and genetic predisposition [2]. Recently, growing studies have focused on the associations of single nucleotide polymorphisms (SNPs) with the risk of CAD, suggesting that genetic polymorphic variations play critical roles on CAD pathogenesis [3, 4]. Long noncoding RNAs (lncRNAs) represent a novel class of regulatory molecule, which are arbitrarily defined as transcripts of more than 200 nucleotides in length that lack protein-coding potential [5]. LncRNAs are involved in the regulation of gene expression, including epigenetic regulation, transcription, and posttranscriptional regulation [6]. In addition, dysregulation of lncRNAs can also contribute to the pathogenesis of diverse human diseases, including CAD [7, 8]. A growing body of evidence suggests that polymorphisms in lncRNAs gene contribute to CAD and myocardial infarction (MI) susceptibility [9–13].

Among these lncRNAs, lincRNA-p21 contains different motifs which can interact with mRNA, RNA-binding proteins, and miRNAs targets [14–16]. LincRNA-p21 is associated with the pathogenesis of human diseases such as prostate cancer, skin tumor, colorectal cancer, hepatocellular carcinoma, chronic lymphocytic leukemia, and rheumatoid arthritis [17–24]. Additionally, lincRNA-p21, a key regulator of vascular smooth muscle cells (VSMCs) proliferation and apoptosis, exerts protective effects against neointima formation in injured carotid arteries [25]. Proliferation of VSMCs and the formation of neointima dominate the atherosclerosis lesion development. Thus, lincRNA-p21 might play a crucial role in the pathogenesis and progression of CAD. Recently, a number of studies have focused on the associations of polymorphic variants in lncRNAs with the risk of CAD [9, 12]. However, whether genetic variants of* lincRNA-p21* contribute to the risk of CAD is still unknown. Therefore, the aim of the present study is to analyze the potential association of* lincRNA-p21* polymorphisms with CAD risk in the Chinese Han population. Our data indicated that the G-A-A-G (rs9380586-rs4713998-rs6930083-rs6931097) haplotype of* lincRNA-p21* gene was associated with decreased risk of CAD, particularly among early-onset CAD or MI subjects (≤60 years old).

## 2. Materials and Methods

### 2.1. Characteristics of Study Subjects

A total of 1270 subjects (615 CAD and 655 CAD-free controls) were recruited from the Affiliated Hospital of Guangdong Medical University and the First People's Hospital of Foshan from March 2011 to October 2014. All study individuals were genetically unrelated Han Chinese. Inclusion and exclusion criteria, diagnosis, and evaluation as well as criteria for CAD, MI, and controls were described previously [4, 26].

The early-onset CAD/MI was defined as those cases with the age of less than 60, according to the previous published studies [27, 28]. Hypertension was defined if the individual's systolic and/or diastolic blood pressure (DBP) was above 140/90 mmHg [29]. Diabetes mellitus, dyslipidemia, and smoking were defined as described in our previous study [26]. This study was approved by the Ethics Committee of the Affiliated Hospital of Guangdong Medical University and the First People's Hospital of Foshan. All recruited subjects ensured their written informed consent to be included in the study regarding the use of their blood specimens for research studies. The structured questionnaires were administered by interviewers to collect medical information at the enrollment.

### 2.2. Biochemical Analysis

Blood samples were collected into test tubes containing EDTA as anticoagulant. The sample was separated at 2000 ×g for 15 min after collection and then stored at −80°C. The levels of total cholesterol (TC), triglyceride (TG), low-density lipoprotein cholesterol (LDL-C), and high-density lipoprotein cholesterol (HDL-C) were determined by an automated chemistry analyzer (Olympus, Japan). The levels of glucose were measured via the glucose oxidase method (Abbott Laboratories, USA).

### 2.3. DNA Extraction

Genomic DNA was extracted from EDTA anticoagulated venous blood according to the instruction of TIANamp blood DNA extraction kit (TianGen Biotech, China). All of the DNA samples were dissolved into water. The final preparation was stored at −20°C.

### 2.4. tagSNP Selection and Genotyping

**Figure 1:**
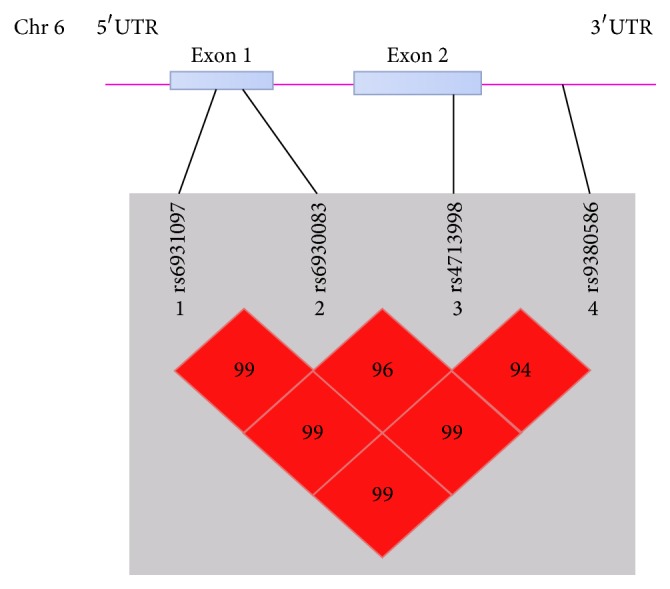
Schematic of* lincRNA-p21* gene structure.* lincRNA-p21* gene is composed of 2 exons which are represented as gray boxes. The lines indicate the locations of single nucleotide polymorphism. *D*′ values are plotted to exhibit LD between the four tagSNPs.

The SNP database of* lincRNA-p21* gene in the Han Chinese population was downloaded from the HapMap Project (http://www.hapmap.org/) [30]. The Haploview software (version 4.2) was prerequisites for tagSNPs selection with minor allele frequency (MAF) larger than 0.05, and linkage disequilibrium (LD) patterns with *r* ^2^ > 0.8 [31]. Totally, four tagSNPs (rs9380586, rs4713998, rs6930083, and rs6931097) were selected for genotyping. The positions of the four tagSNPs were shown in Figure 1. The *r* ^2^ information for the four tagSNPs and alleles captured accordingly was exhibited in Table S1 in Supplementary Material available online at http://dx.doi.org/10.1155/2016/9109743. The haplotypic blocks of the four tagSNPs were estimated by the Haploview software. Then haplotype analysis was performed through the SHEsis software (http://analysis.bio-x.cn/myAnalysis.php) [32].

Genomic DNA was genotyped using PCR-ligase detection reaction method (PCR-LDR) (Shanghai Biowing Applied Biotechnology Company). These sequences of probes and primers were shown in Table S2. The PCR reaction (20 *μ*L) contained 50 ng genomic DNA, 0.5 *μ*M primer mix, 1x PCR buffer, 1 U Taq polymerase, 2 mM dNTPs, and 3 mM MgCl_2_ on the Gene Amp PCR system 9600 (Norwalk, USA). The cycling parameters were carried out as the following procedure: 95°C for 2 min; 40 cycles at 94°C for 90 s, 56°C for 90 s, and 65°C for 30 s; and a final extension step at 65°C for 10 min. The ligation reaction for PCR product was done in a 10 *μ*L volume containing 1 *μ*L 1x buffer, 2 pmol probe mix, 2 U Taq DNA ligase, and 4 *μ*L of PCR product. The LDR parameters were as the following procedure: 95°C for 2 min and 40 cycles at 94°C for 15 s and 50°C for 25 s. Then, 1 *μ*L LDR reaction product was added into 1 *μ*L loading buffer and 1 *μ*L ROX. The mixture was then analyzed by the ABI Prism 3730 DNA Sequencer (Applied Biosystems, USA). For ten percent of samples, the PCR was repeated once for quality control. Repeatability of results was 100%.

### 2.5. Statistical Analysis

Data analysis was performed by using the SPSS computer software (version 21). The haplotype analysis was undertaken using the SHEsis online webserver. Each tagSNP was tested in controls for confirmation with Hardy-Weinberg Expectations (HWE) by a goodness-of-fit *χ* ^2^ test. Continuous variables between the CAD/MI and control groups were presented as means ± standard deviation and compared using Student's* t*-test. *χ* ^2^ test was used for categorical variables in the differences of the demographic characteristics at the CAD/MI and control groups. Logistic regression analysis was used to analyze the association between the risk for CAD/MI and the tagSNPs with adjustment for risk factors of CAD (sex, age, smoking, drinking, diabetes, hyperlipidemia, and hypertension).* P* < 0.05 was considered to indicate statistical significance.

## 3. Results

### 3.1. Characteristics of Study Subjects

The clinical features of individuals recruited in this study were listed in Table 1 and Table S3. The levels of TG and LDL-C were significantly higher in the CAD or MI patients than in the controls (all* P* < 0.001). The patients with CAD or MI had higher levels of diastolic blood pressure, systolic blood pressure, and lower levels of HDL-C (all* P* < 0.001). There were more smokers and drinkers in the CAD or MI cases than in the controls. There was a higher prevalence of subjects with hyperlipidemia, hypertension, or diabetes among the CAD or MI patients. Additionally, the number of male individuals in the CAD or MI cases was much higher than the female individuals. These data showed that alcohol intake, smoking, hyperlipidemia, hypertension, diabetes mellitus, and male gender were the crucial risk factors for developing CAD/MI in Chinese population.

### 3.2. Multivariate Associations of* lincRNA-p21 *tagSNPs with the Risk of CAD

Four* lincRNA-p21* tagSNPs (rs9380586, rs4713998, rs6930083, and rs6931097) were genotyped in CAD (*n* = 615) and control subjects (*n* = 655). The primary information for these polymorphisms was exhibited in Table 2. The observed genotype distribution of four tagSNPs in the controls conformed to the Hardy-Weinberg equilibrium (all* P* values ≥ 0.05, Table 2), providing no evidence of population stratification within the dataset. To define whether there was a statistically significant increased risk of CAD susceptibility, we conducted an analysis using a logistic regression model, after adjustment for traditional risk factors of CAD and MI (age, gender, drinking, smoking, hypertension, hyperlipidemia, and diabetes; Table 3). No association of these tagSNPs with CAD risk was detected in allelic or genotypic analyses (Table 3). Additionally, there is also no significant difference for allele frequencies and genotype distributions among premature CAD or MI subjects (Table S4).

### 3.3. Association between the Haplotypes of* lincRNA-p21* tagSNPs with the Risk of CAD

Haplotype analyses were carried out to further investigate the combinational effects of these four polymorphisms on CAD risk. As shown in Figure 1, four tagSNPs were located within one haplotypic block. Five common haplotypes with frequency > 3% accounted for almost all of the haplotype variations (Table 4). We further compared the haplotype frequencies between CAD and controls. Among the five common haplotypes, only the haplotype G-A-A-G (rs9380586-rs4713998-rs6930083-rs6931097) was found to be associated with a decreased risk for CAD (OR = 0.78, 95% CI = 0.63–0.97,* P* = 0.023, Table 4). MI is one of the major adverse outcomes of CAD. Further analysis showed that haplotype G-A-A-G had a significantly lower risk for MI (OR = 0.68, 95% CI = 0.51–0.91,* P* = 0.010, Table 4). Premature CAD or MI is known to be the most aggressive form of the disease given no early signs and symptoms. In this study, the decreased risk was more pronounced among premature CAD and MI subjects (≤60 years old) carrying haplotype G-A-A-G (*P* = 0.017, OR = 0.67 for premature CAD, and* P *= 0.041, OR = 0.65 for premature MI, Table 5).

## 4. Discussion

Growing evidences have demonstrated that* lincRNA-p21* may participate in the pathogenesis and progression of CAD [25]. However, the biological significance of the* lincRNA-p21* polymorphisms on CAD risk is undetermined. In this study, we evaluated the association between* lincRNA-p21* tagSNPs and the risk of CAD/MI in the Chinese Han population. We found that the haplotype G-A-A-G was associated with decreased risk of CAD and MI, particularly among premature CAD/MI in Chinese Han population. Our data provided the first evidence that the haplotype G-A-A-G of* lincRNA-p21* may be involved in susceptibility to CAD or MI.

Previous investigations have demonstrated that genetic variants in lncRNA gene affect CAD/MI susceptibility. For example, Ishii et al. identified SNPs in* MIAT* conferring susceptibility to MI [12]. Polymorphisms in lncRNA* ANRIL* have been found to be associated with the risk of MI [13]. In the present case-control study, we identified that the haplotype G-A-A-G of* lincRNA-p21* correlates with the decreased risk of CAD/MI in a Chinese population, suggesting that the haplotype G-A-A-G could be a protective factor for CAD/MI. CAD may be affected by changes related to the function of this genomic region, rather than individual loci* per se*. There is a possibility that the haplotype is a causal variant, by regulating the gene expression of* lincRNA-p21*, and subsequently contributes to the risk of CAD/MI. The precise mechanisms regarding how the haplotype G-A-A-G of* lincRNA-p21* affects CAD risk need further investigation.

Our stratified analyses revealed that the decreased risk of haplotype G-A-A-G in premature CAD and MI subjects (≤60 years old) was more evident. Relative high-level exposure to environmental risk factors and weak immune system in older subjects may account for these. These potential risks of CAD in older individuals are more likely because of the aging effects instead of the direct genetic effects. Therefore, the* lincRNA-p21* haplotype may be more influential in premature CAD/MI.

Several limitations need to be acknowledged in this study. First, our study was performed in a Chinese Han population, and the data should be extrapolated to other regions and ethnic groups cautiously. Second, the sample size of the present study was relatively small. Therefore, enlarging the sample size would make the conclusions more convincing. Finally, further studies in different population may contribute to establish the true significance of the association between this haplotype and the risk of CAD/MI. However, our data provided valuable insights and intriguing information in the development of CAD.

## 5. Conclusions

In summary, our findings indicate that the G-A-A-G haplotype of the* lincRNA-p21* gene is associated with a significantly decreased risk of CAD/MI, and the effects are more significant among younger subjects (≤60 years old). These results suggest that the G-A-A-G haplotype may contribute to the risk of CAD/MI, which may be translated into improved CAD/MI risk assessment and prevention. However, further studies with more subjects and in diverse ethnic populations are warranted to clarify the general validity of our finding.

## Supplementary Material

The information for alleles captured by rs9380586, rs4713998, rs6930083 and rs6931097 was shown in TABLE S1. The sequences of the primers and probes used to genotype the rs9380586, rs4713998, rs6930083 and rs6931097 polymorphisms were shown in TABLE S2. The characteristics of premature CAD, MI and controls were shown in TABLE S3. Multivariate associations of the SNPs in lincRNA-p21 gene with the risk of premature CAD/premature MI were shown in TABLE S4.

## Figures and Tables

**Table 1:** The characteristics of CAD, MI, and controls.

Variable	Controls (*n* = 655)	CAD (*n* = 615)	MI (*n* = 279)	*P* ^a^ versus controls	
				CAD	MI
Age (years)	61.50 ± 12.50	63.85 ± 11.71	62.03 ± 11.90	0.001^b^	0.552
Sex (male)	379 (57.9%)	431 (70.1%)	217 (77.8%)	**<0.001**	**<**0.001^b^
Smoking	154 (23.5%)	341 (55.4%)	166 (59.5%)	**<0.001**	**<0.001**
Drinking	99 (15.1%)	143 (23.3%)	72 (25.8%)	**<0.001**	**<0.001**
Hypertension	221 (33.7%)	377 (61.3%)	171 (61.3%)	**<0.001**	**<0.001**
Diabetes	104 (15.9%)	270 (43.9%)	127 (45.5%)	**<0.001**	**<0.001**
Hyperlipidemia	239 (36.5%)	423 (68.8%)	196 (70.3%)	**<0.001**	**<0.001**
Systolic BP (mm Hg)	131.83 ± 19.38	141.06 ± 18.19	139.53 ± 19.17	**<0.001**	**<0.001**
Diastolic BP (mm Hg)	72.78 ± 10.63	76.29 ± 10.45	75.78 ± 11.48	**<0.001**	**<0.001**
FPG (mM)	5.79 ± 1.95	6.58 ± 1.63	6.57 ± 1.76	**<0.001**	**<0.001**
Triglycerides (mM)	1.48 ± 0.81	2.05 ± 1.02	2.08 ± 1.01	**<0.001**	**<0.001**
Total cholesterol (mM)	4.59 ± 1.15	4.70 ± 1.24	4.75 ± 1.23	0.099	0.062
HDL cholesterol (mM)	1.39 ± 0.67	1.19 ± 0.38	1.20 ± 0.44	**<0.001**	**<0.001**
LDL cholesterol (mM)	2.61 ± 0.92	3.02 ± 0.92	3.07 ± 0.99	**<0.001**	**<0.001**

^a^Two-sided chi-square test or independent-samples *t*-test.

^b^ *P* values under 0.05 were shown in bold font.

**Table 2:** Primary information for the polymorphisms in *lincRNA-p21* gene.

Genotyped SNPs	rs9380586	rs4713998	rs6930083	rs6931097
Chr Pos (Genome Build 106)	36663787	36664833	36666379	36666682
Pos in *lincRNA-p21* gene	3′ UTR	Exon 2	Exon 1	Exon 1
MAF^a^ for Chinese population (CHB) in HapMap	0.120	0.280	0.230	0.430
MAF in controls (*n* = 655)	0.102	0.210	0.274	0.390
*P* value for HWE^b^ test in controls	0.335	0.839	0.158	0.956

^a^MAF: minor allele frequency.

^b^HWE: Hardy-Weinberg equilibrium.

**Table 3:** Multivariate associations of four SNPs in *lincRNA-p21* gene with the risk of CAD or MI.

Type	Controls	Cases		OR (95% CI)^a^ versus controls		*P* ^a^ versus controls	
	Number (%)	CAD (%)	MI (%)	CAD	MI	CAD	MI
	*n* = 655	*n* = 615	*n* = 279				
*rs9380586*							
G	1177 (89.8)	1104 (89.8)	496 (88.9)	1	1		
A	133 (10.2)	126 (10.2)	62 (11.1)	0.97 (0.72–1.32)	1.12 (0.77–1.64)	0.856	0.559
GG	531 (81.1)	497 (80.8)	223 (79.9)	1	1		
AA+AG	124 (18.9)	118 (19.2)	56 (20.1)	1.00 (0.71–1.39)	1.01 (0.66–1.54)	0.986	0.981
*rs4713998*							
A	1035 (79.0)	960 (78.0)	431 (77.2)	1	1		
G	275 (21.0)	270 (22.0)	127 (22.8)	1.05 (0.84–1.31)	1.12 (0.77–1.64)	0.686	0.231
AA	408 (62.3)	374 (60.8)	164 (58.8)	1	1		
AG+GG	247 (37.7)	241 (39.2)	115 (41.2)	1.01 (0.77–1.32)	1.01 (0.66–1.54)	0.959	0.336
*rs6930083*							
G	951 (72.6)	931 (75.7)	425 (76.2)	1	1		
A	359 (27.4)	299 (24.3)	133 (23.8)	0.86 (0.69–1.07)	0.83 (0.62–1.10)	0.177	0.200
GG	338 (51.6)	349 (56.7)	160 (57.3)	1	1		
AG+AA	317 (48.4)	266 (43.3)	119 (42.7)	0.82 (0.63–1.07)	0.79 (0.56–1.11)	0.145	0.172
*rs6931097*							
G	799 (61.0)	719 (58.5)	327 (58.6)	1	1		
A	511 (39.0)	511 (41.5)	231 (41.4)	1.12 (0.93–1.35)	1.15 (0.89–1.47)	0.246	0.286
GG	244 (37.3)	204 (33.2)	91 (32.6)	1	1		
AA+AG	411 (62.7)	411 (66.8)	188 (67.4)	1.16 (0.88–1.52)	1.19 (0.83–1.69)	0.297	0.347

^a^Adjusted for sex, age, smoking, drinking, hyperlipidemia, hypertension, and diabetes.

**Table 4:** Association between *lincRNA-p21* gene haplotypes with the risk of CAD or MI.

Haplotype^a^	Controls	Cases	OR (95% CI)	*P*
	Number (%)	Number (%)		
CAD	*n* = 655	*n* = 615		

AAAG	132.99 (10.2)	124.70 (10.1)	1.00 (0.77–1.30)	0.994
GAAG	225.98 (17.3)	171.45 (13.9)	0.78 (0.63–0.97)	0.023^b^
GAGA	235.99 (18.0)	242.27 (19.7)	1.12 (0.92–1.37)	0.266
GAGG	440.03 (33.6)	421.58 (34.3)	1.04 (0.88–1.22)	0.685
GGGA	274.98 (21.0)	267.15 (21.7)	1.05 (0.87–1.27)	0.633

MI	*n* = 655	*n* = 279		

AAAG	132.99 (10.2)	60.70 (10.9)	1.09 (0.79–1.50)	0.611
GAAG	225.98 (17.3)	69.17 (12.4)	0.68 (0.51–0.91)	0.010^b^
GAGA	235.99 (18.0)	105.28 (18.9)	1.07 (0.83–1.38)	0.624
GAGG	440.03 (33.6)	195.85 (35.1)	1.08 (0.88–1.33)	0.477
GGGA	274.98 (21.0)	123.87 (22.2)	1.08 (0.85–1.38)	0.521

^a^The allelic sequence in the haplotypes is in the following order: rs9380586, rs4713998, rs6930083, rs6931097. Haplotype with frequency less than 3% was pooled and not analyzed.

^b^ *P* values under 0.05 were shown in bold font.

**Table 5:** Association between *lincRNA-p21* gene haplotypes with the risk of premature CAD/MI.

Haplotype^a^	Controls	Cases	OR (95% CI)	*P*
	Number (%)	Number (%)		
Premature CAD	*n* = 309	*n* = 238		

AAAG	63.00 (10.2)	56.77 (11.9)	1.20 (0.82–1.76)	0.344
GAAG	118.99 (19.3)	65.29 (13.7)	0.67 (0.48–0.93)	0.017^b^
GAGA	111.00 (18.0)	95.23 (20.0)	1.15 (0.85–1.56)	0.364
GAGG	193.01 (31.2)	167.71 (35.2)	1.21 (0.94–1.56)	0.142
GGGA	131.99 (21.4)	88.06 (18.5)	0.84 (0.62–1.14)	0.263

Premature MI	*n* = 309	*n* = 129		

AAAG	63.00 (10.2)	29.68 (11.5)	1.17 (0.73–1.85)	0.514
GAAG	118.99 (19.3)	34.16 (13.2)	0.65 (0.43–0.99)	**0.041**
GAGA	111.00 (18.0)	44.31 (17.2)	0.97 (0.66–1.42)	0.859
GAGG	193.01 (31.2)	96.85 (37.5)	1.36 (1.00–1.84)	**0.049**
GGGA	131.99 (21.4)	48.84 (18.9)	0.88 (0.61–1.27)	0.484

^a^The allelic sequence in the haplotypes is in the following order: rs9380586, rs4713998, rs6930083, rs6931097. Haplotype with frequency less than 3% was pooled and not analyzed.

^b^ *P* values under 0.05 were shown in bold font.
