# Supplementary material for: Association of lincRNA-p21 Haplotype with Coronary Artery Disease in a Chinese Han Population
Source: Dis Markers. 2016 Jun 2;2016:9109743. doi: 10.1155/2016/9109743 (PMC4909913; doi:10.1155/2016/9109743)
Supplement: Supplementary file 1 — The information for alleles captured by rs9380586, rs4713998, rs6930083 and rs6931097 was shown in TABLE S1. The sequences of the primers and probes used to genotype the rs9380586, rs4713998, rs6930083 and rs6931097 polymorphisms were shown in TABLE S2. The characteristics of premature CAD, MI and controls were shown in TABLE S3. Multivariate associations of the SNPs in lincRNA-p21 gene with the risk of premature CAD/premature MI were shown in TABLE S4. [file 9109743.f1.pdf]

TABLE S1: The information for alleles captured by rs9380586, rs4713998, rs6930083 and rs6931097, accordingly.

Alleles captured	Location	tagSNP	r^2
rs6930083	Exon 1	rs6930083	1.0
rs4711457	Exon 2	rs6930083	1.0
rs4713999	Exon 2	rs6930083	1.0
rs9394368	3' UTR	rs6930083	1.0
rs4713996	3' UTR	rs6930083	1.0
rs4714001	5' UTR	rs6930083	0.938
rs4713997	3' UTR	rs9380586	1.0
rs9380586	3' UTR	rs9380586	1.0
rs9380585	3' UTR	rs9380586	1.0
rs4714000	5' UTR	rs9380586	1.0
rs6931097	Exon 1	rs6931097	1.0
rs4713998	Exon 2	rs4713998	1.0

TABLE S2. The sequences of the primers and probes used to genotype the rs9380586, rs4713998, rs6930083 and rs6931097 polymorphisms.

Name	Sequence (5'-3')
**Primers**	
rs9380586-F	GGAGCCTTTCTATAGTTTTC
rs9380586-R	AATCAATGAATCCTCCTCTG
rs4713998-F	AGAATAACCCGAGCTGAAGG
rs4713998-R	ATGCATAGACGGTTGTGGTG
rs6930083-F	AAGGATGTGAAGCCATTTGC
rs6930083-R	GTGAGCAGTCAGCCAGTTTG
rs6931097-F	AGAAAATGGGTAGCTGGAGG
rs6931097-R	CATTCACATGGTCCACAGTC
**Probes**	
rs9380586_modify	P-CCTTTTCATGATATTAAAAATTTTTTTTTTTTTTTTTTTTTT-FAM
rs9380586_A	TTTTTTTTTTTTTTTTTTTTAATGAATCCTCCTCTGAAACAAT
rs9380586_G	TTTTTTTTTTTTTTTTTTTTTTAATGAATCCTCCTCTGAAACAAC
rs4713998_modify	P-AGAGAGAGAATGGGGCCATCTTTTTTTTTTTTTTTTTTTTTTTTTT<br>TTTTTTTT-FAM
rs4713998_A	TTTTTTTTTTTTTTTTTTTTTTTTTTTTTTTTGGAGGACACAGGAGA<br>GGCAAGAT
rs4713998_G	TTTTTTTTTTTTTTTTTTTTTTTTTTTTTTTTTTGGAGGACACAGGA<br>GAGGCAAGAC
rs6930083_modify	P-GCTAGCTGTGGGCTGTAATGTTTTTTTTTTTTTTTTTTTTTTTTTT<br>TTTT-FAM
rs6930083_A	TTTTTTTTTTTTTTTTTTTTTTTTTTTTGGCAAACCAAGACAGTAGG<br>TCCT
rs6930083_G	TTTTTTTTTTTTTTTTTTTTTTTTTTTTTTGGCAAACCAAGACAGTA<br>GGTCCC
rs6931097_modify	P-CTCCTACCCCTACCCTCCAGTTTTTTTTTTTTTTTTT-FAM
rs6931097_A	TTTTTTTTTTTTTTTTAGGCTATTCAGTGAAAACTCTCT
rs6931097_G	TTTTTTTTTTTTTTTTTTAGGCTATTCAGTGAAAACTCTCC

TABLE S3. The characteristics of premature CAD, MI and controls.

Variable	Controls (n =309)	CAD (n =238)	MI (n =129)	*P* [a] vs. controls	
				**CAD**	**MI**
Age (years)	50.47 ± 6.96	51.77 ± 6.40	51.40 ± 6.29	**0.025** [b]	0.194
Sex (male)	175 (56.6%)	189 (79.4%)	109 (85.4%)	**<0.001**	**<0.001**[b]
Smoking	83 (26.9%)	150 (63.0%)	84 (65.1%)	**<0.001**	**<0.001**
Drinking	50 (16.2%)	69 (29.0%)	36 (27.9%)	**<0.001**	**0.005**
Hypertension	76 (24.6%)	148 (62.2%)	78 (60.5%)	**<0.001**	**<0.001**
Diabetes	44 (14.2%)	113 (47.5%)	62 (48.1%)	**<0.001**	**<0.001**
Hyperlipidemia	125 (40.5%)	176 (73.9%)	97 (75.2%)	**<0.001**	**<0.001**
Systolic BP (mm Hg)	128.50 ± 15.79	141.83 ± 18.76	140.13 ± 19.91	**<0.001**	**<0.001**
Diastolic BP (mm Hg)	72.83 ± 10.10	76.97 ± 10.42	76.21 ± 11.27	**<0.001**	**0.002**
FPG (mM)	5.78 ± 2.32	6.59 ± 1.60	6.61 ± 1.64	**<0.001**	**<0.001**
Triglycerides (mM)	1.58 ± 0.94	2.16 ± 1.08	2.17 ± 1.11	**<0.001**	**<0.001**
Total cholesterol (mM)	4.61 ± 1.18	4.83 ± 1.25	4.88 ± 1.19	**0.041**	**0.036**
HDL cholesterol (mM)	1.44 ± 0.89	1.19 ± 0.38	1.15 ± 0.38	**<0.001**	**<0.001**
LDL cholesterol (mM)	2.61 ± 0.99	3.08 ± 0.93	3.12 ± 0.98	**<0.001**	**<0.001**

[a] Two-sided chi-square test or independent-samples *t*-test.
[b] *P* values under 0.05 were shown in bold font.

TABLE S4. Multivariate associations of the SNPs in *lincRNA-p21* gene with the risk of premature CAD/MI.

Type	Controls	premature Cases		OR (95% CI) [a] vs. controls		*P* [a] vs. controls	
	No. (%) n=309	CAD (%) n =238	MI (%) n =129	CAD	MI	CAD	MI
rs9380586							
G	555 (89.8)	418 (87.8)	227 (88.0)	1	1		
A	63 (10.2)	58 (12.2)	31 (12.0)	1.40 (0.86-2.28)	1.42 (0.79-2.57)	0.172	0.244
GG	251 (81.2)	184 (77.3)	100 (77.6)	1	1		
AA+AG	58 (18.8)	54 (22.7)	29 (22.4)	1.38 (0.81-2.36)	1.33 (0.69-2.54)	0.235	0.393
rs4713998							
A	486 (78.6)	385 (80.9)	205 (79.5)	1	1		
G	132 (21.4)	91 (19.1)	53 (20.5)	0.77 (0.52-1.14)	0.89 (0.55-1.44)	0.187	0.634
AA	191 (61.8)	155 (65.1)	79 (61.2)	1	1		
AG+GG	118 (38.2)	83 (34.9)	50 (38.8)	0.70 (0.45-1.10)	0.88 (0.51-1.51)	0.125	0.646
rs6930083							
G	436 (70.6)	351 (73.7)	205 (79.5)	1	1		
A	182 (29.4)	125 (26.3)	53 (20.5)	0.94 (0.66-1.35)	1.00 (0.64-1.56)	0.743	0.997
GG	149 (48.2)	127 (53.4)	79 (61.2)	1	1		
AG+AA	160 (51.8)	111 (46.6)	50 (38.8)	0.90 (0.59-1.39)	1.02 (0.60-1.73)	0.646	0.942
rs6931097							
G	375 (60.7)	291 (61.1)	162 (62.8)	1	1		
A	243 (39.3)	185 (38.9)	96 (37.2)	0.89 (0.67-1.25)	0.86 (0.58-1.27)	0.583	0.434
GG	115 (37.2)	86 (36.1)	48 (37.2)	1	1		
AA+AG	194 (62.8)	152 (63.9)	81 (62.8)	0.92 (0.59-1.43)	0.87 (0.51-1.50)	0.708	0.618

[a] Adjusted for sex, smoking, drinking, hypertension, diabetes, hyperlipidemia.
